# Supplementary material for: Detection of Bioactive Peptides’ Signature in Podolica Cow’s Milk
Source: Foods. 2025 Mar 4;14(5):877. doi: 10.3390/foods14050877 (PMC11898676; doi:10.3390/foods14050877)
Supplement: Supplementary file 1 [file foods-14-00877-s001.zip › foods-3501111-supplementary.pdf]

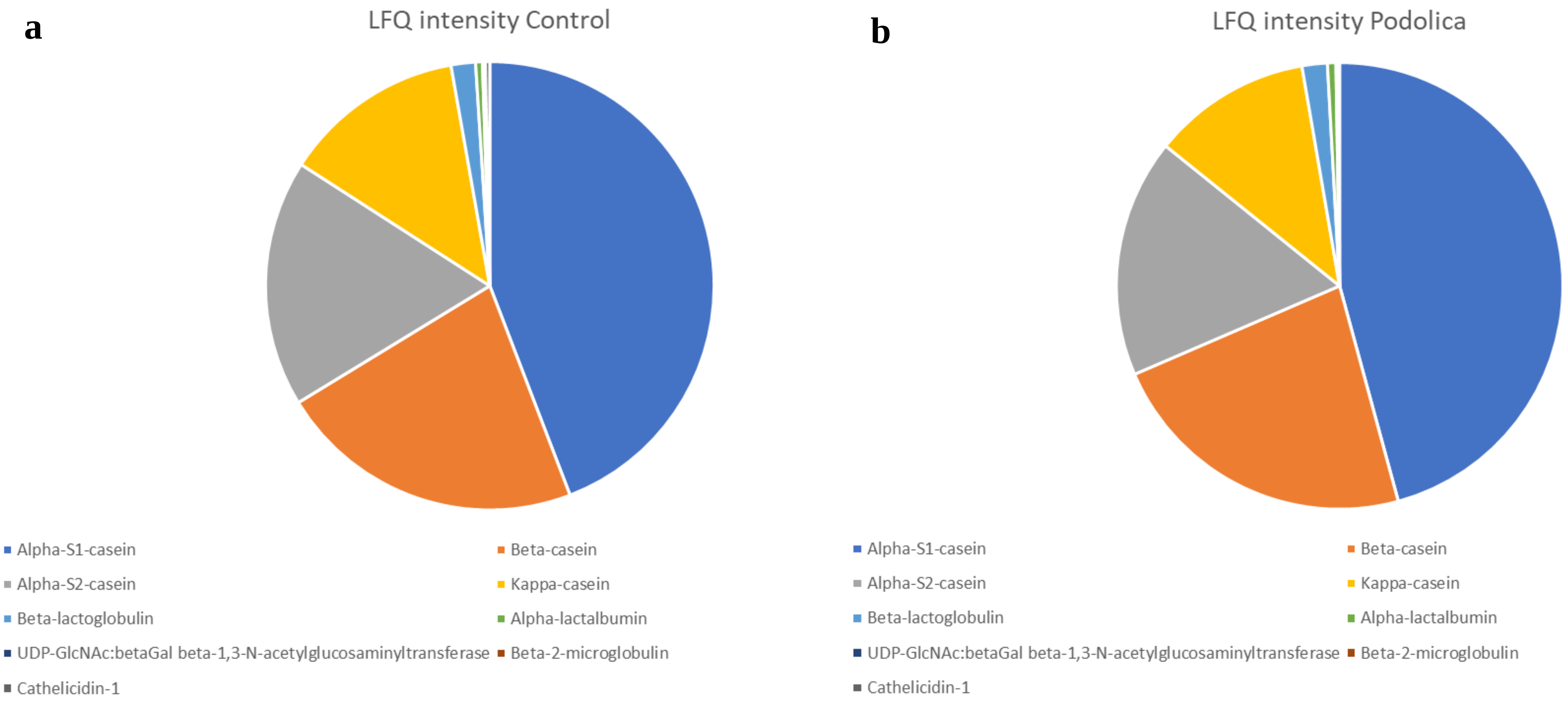
a
LFQ intensity Control
Alpha-S1-casein
Beta-casein
Alpha-S2-casein
Kappa-casein
Beta-lactoglobulin
Alpha-lactalbumin
UDP-GlcNAc:betaGal beta-1,3-N-acetylglucosaminyltransferase
Beta-2-microglobulin
Cathelicidin-1
b
LFQ intensity Podolica
Alpha-S1-casein
Beta-casein
Alpha-S2-casein
Kappa-casein
Beta-lactoglobulin
Alpha-lactalbumin
UDP-GlcNAc:betaGal beta-1,3-N-acetylglucosaminyltransferase
Beta-2-microglobulin
Cathelicidin-1
